# Metabolic Engineering of *Rhodotorula toruloides* for Biosynthesis of Retinal

**DOI:** 10.3390/jof12040258

**Published:** 2026-04-02

**Authors:** Huihui Qiu, Linyue Tian, Lin Hu, Lianwu Wu, Yu Huang, Ran Ge, Yufan Xing, Alexander A. Kamnev, Ning He, Mingfeng Cao

**Affiliations:** 1 Department of Chemical and Biochemical Engineering, College of Chemistry and Chemical Engineering, Key Laboratory for Synthetic Biotechnology of Xiamen City, Xiamen University, Xiamen 361005, China; 20620240156801@stu.xmu.edu.cn (H.Q.); tly3110570@163.com (L.T.); hulin@stu.xmu.edu.cn (L.H.); lianwuwu@stu.xmu.edu.cn (L.W.); yuhuang_0012@163.com (Y.H.); geran@stu.xmu.edu.cn (R.G.); yufan18888@163.com (Y.X.); 2 Innovation Laboratory for Sciences and Technologies of Energy Materials of Fujian Province (IKKEM), Xiamen 361005, China; 3 Institute of Biochemistry and Physiology of Plants and Microorganisms, Saratov Federal Scientific Center of the Russian Academy of Sciences, Saratov 410049, Russia; a.a.kamnev@mail.ru

**Keywords:** *Rhodotorula toruloides*, β-carotene, retinal, metabolic engineering, fed-batch fermentation

## Abstract

Rapid advancements in biotechnology have enabled biomanufacturing to emerge as a feasible approach for industrial chemical production. By harnessing synthetic biology and metabolic engineering, engineered microbial cell factories can convert renewable resources into valuable chemicals, providing a sustainable alternative to traditional chemical methods. This study focuses on the microbial production of retinal, an important retinoid used in pharmaceuticals, food, and cosmetics. The oleaginous yeast *Rhodotorula toruloides* NP11 was genetically modified to synthesize retinal by incorporating and optimizing three β-carotene 15,15′-dioxygenase genes from various sources. Several genetic modifications were made to enhance retinal yield, including the overexpression of isopentenyl-diphosphate isomerase (IDI1), geranylgeranyl diphosphate synthase (BTS1), phytoene synthase (CARRP), and phytoene dehydrogenase (CARB), which led to increased β-carotene levels and boosted retinal production. Furthermore, fermentation conditions such as temperature, antioxidants, and extractants were fine-tuned. The engineered strain Rt13 ultimately achieved a maximum retinal concentration of 20.38 mg/L through fed-batch fermentation. This study highlights the potential of *R*. *toruloides* as a cell factory for terpenoid biosynthesis, providing valuable insights for future metabolic engineering endeavors.

## 1. Introduction

Recent advancements in biotechnology indicate that biomanufacturing could emerge as a new approach to industrial chemical production, utilizing synthetic biology to create cell factories that transform renewable resources into valuable chemicals through metabolic pathways [1,2,3]. This eco-friendly manufacturing method has garnered significant attention from those dedicated to environmental protection and sustainable development. Utilizing microorganisms like yeast or bacteria for chemical production streamlines the complex processes of chemical synthesis and allows for the creation of various natural compounds [4,5]. Terpenoids are widely studied in microbial synthesis due to their structural diversity, functional variety, and significant biological activities [6,7]. Additionally, progress in metabolic engineering has facilitated the efficient creation of valuable terpenoids, positioning microbial synthesis as a viable solution for industrial uses in pharmaceuticals, fragrances, and biofuels [8,9,10].

Retinal, the aldehyde derivative of vitamin A, is a crucial retinoid present in the sensory cells of the retina, playing a vital role in the visual cycle [11]. Its exceptional optical characteristics and chemical reactivity render it valuable for a variety of applications [12]. Consequently, retinal is extensively used in medicine, food, and cosmetics, where its ability to interact with biological systems and light provides significant advantages [13,14]. Retinal can be produced through natural extraction, chemical synthesis, or biosynthesis. In the chemical synthesis process, retinol is initially generated and then oxidized to form retinal or retinoic acid [15]. However, this method is difficult to control and frequently leads to a mix of retinoids. While chemical synthesis remains the main approach for producing retinoids, it necessitates laborious downstream separation and purification, which, coupled with the demand for more sustainable practices, limits its advancement.

The key step in retinal biosynthesis is the catalysis by β-carotene 15,15′-dioxygenase (BCO), a major rate-limiting enzyme that cleaves one molecule of β-carotene into two molecules of retinal [16]. Yeast is an ideal candidate for terpenoid production because of its robustness and naturally efficient mevalonate (MVA) pathway [17]. A strain of *Saccharomyces cerevisiae* was engineered to produce 3350 mg/L of vitamin A through fed-batch fermentation, yielding 2094 mg/L of retinal and 1256 mg/L of retinol from xylose [18]. The conversion of retinal to retinol or retinoic acid by oxidoreductases in vivo poses a significant challenge for retinal production [19]. To address this, another *S*. *cerevisiae* strain was developed by deleting alcohol and aldehyde dehydrogenases, resulting in a high-purity retinal yield of 69.13 mg/L via the heterologous expression of BCO from a marine bacterium [20]. Additionally, a previous study showed that introducing a heterologous MVA pathway into *Escherichia coli*, alongside its native MEP pathway, enhanced the precursor supply, leading to the synthesis of 136 mg/L of retinoids, including 67 mg/L of retinal after 72 h [21]. To increase the retinol content, they overexpressed the *ybbO* gene and knocked out the *cat* gene, achieving 88% retinol composition [22]. Furthermore, β-carotene 15,15′-monooxygenase from *Mus musculus* was expressed and purified in *E*. *coli*, facilitating the conversion of β-carotene to retinal, resulting in 72 mg/L of retinal produced in 15 h with a 36% conversion yield after optimization [23]. This study highlights a promising pathway for the in vitro enzymatic biocatalysis of retinal.

*Rhodotorula toruloides* (also known as *Rhodosporidium toruloides*) is a nonconventional basidiomycetous yeast that naturally accumulates significant quantities of lipids and carotenoids. As an oleaginous yeast, it is considered a promising microbial platform for producing valuable biofuels and chemicals [24,25]. *R*. *toruloides* can utilize a wide range of substrates, efficiently metabolizing traditional carbon sources such as glucose, xylose, arabinose, and acetate, along with cost-effective materials like lignocellulosic hydrolysates, vegetable waste, crude glycerol, and industrial wastewater [26]. It also exhibits strong tolerance to osmotic stress, low pH, and high temperatures [27,28]. Recent research has increasingly focused on reconstructing its metabolic network for chemical production and developing genetic tools for genome engineering. The Zhao group successfully mapped the entire central metabolic pathway of *R*. *toruloides* using genomic and transcriptomic data, annotating 8171 protein-coding genes and clarifying the metabolic pathways for lipids and carotenoids [29]. That study has facilitated advanced metabolic engineering efforts aimed at producing terpenoids, particularly carotenoids. For instance, Lin et al. created a mutant library of *R*. *toruloides* transformants with diverse colors through *Agrobacterium tumefaciens*-mediated transformation (ATMT), allowing them to identify mutants with varying growth traits and carotenoid profiles [30]. Zhuang et al. explored the potential of *R*. *toruloides* as a host for terpenoid production, assessing the efficacy of different terpene synthases in generating monoterpenes [31]. They achieved a notable yield of 34.6 mg/L of 1,8-cineole from lignocellulosic biomass in a 2-L batch fermentation by optimizing terpene synthases. The same group further engineered *R. toruloides* for the production of 1,8-cineole and α-bisabolene, achieving titers of 2.6 g/L and 1.4 g/L, respectively, after optimizing enzyme overexpression and promoter modifications [32]. *R. toruloides* is inherently rich in acetyl-CoA reserves due to its robust lipid production capabilities and thus has been frequently utilized for the synthesis of acetyl-CoA-derived compounds. The 2-pyrone synthase gene from *Gerbera hybrida* (*GhPS*), ATP-citrate lyase (*ACL1*), and acetyl-CoA carboxylase (*ACC1*) were in turn introduced into *R. toruloides* IFO0880, which realized an excellent production of triacetic acid lactone [33]. A fatty acyl-CoA reductase from *Marinobacter aqueolei* was inserted randomly into the genomic DNA of *R. toruloides* IFO0880. A series of gene overexpressions and CRISPR/Cas9-mediated gene deletions were implemented, resulting in an increase in fatty alcohol production to 3.7 g/L [34]. The increase in acetyl-CoA and malonyl-CoA pools was achieved by overexpressing *ACL1* and *ACC1* in both of the studies mentioned above.

**Figure 1:**
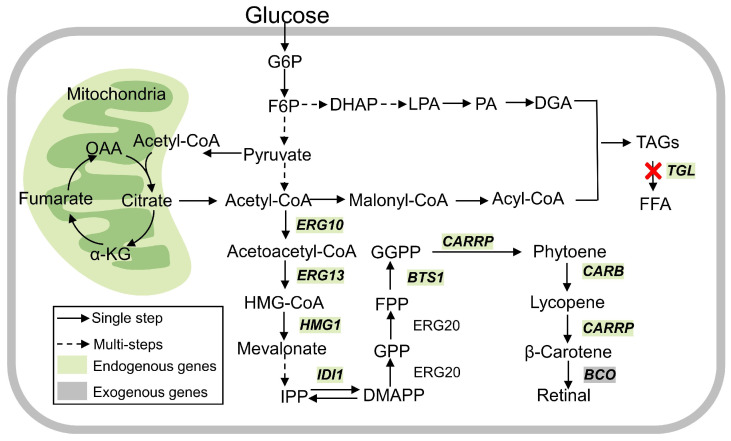
Metabolic pathway for biosynthesis of retinal in *R. toruloides*. These genes are organized into three functional modules: (i) the MVA module, comprising *ERG10*, *ERG13*, *HMG1*, and *IDI1* for enhanced precursor supply; (ii) the carotenoid module, including *BTS1*, *CARRP*, and *CARB* for β-carotene synthesis; and (iii) the retinal module, consisting of the *BCO* for the oxidative cleavage of β-carotene into retinal. G6P, glucose-6-phosphate; F6P, fructose-6-phosphate; DAHP, 3-deoxy-arabino-heptulonate-7-phosphate; LPA, lysophosphatidic acid; PA, phosphatidic acid; DAG, diacylglycerol; TAG, triacylglycerol; FFA, free fatty acid; ERG10, acetoacetyl-CoA thiolase; ERG13, hydroxymethylglutaryl-CoA synthase; HMG1, hydroxymethylglutaryl-CoA reductase; IDI1, isoprene diphosphate isomerase; ERG20, bifunctional FPP synthase; BTS1, geranylgeranyl diphosphate synthase; BCO, β-carotene 15,15′-dioxygenase; CARRP, bifunctional lycopene cyclase/phytoene synthase; CARB, phytoene dehydrogenase; TGL, triacylglycerol lipase; HMG-CoA, hydroxymethylglutaryl-CoA; IPP, isopentenyl pyrophosphate; DMAPP, dimethylallyl diphosphate; GPP, geranyl pyrophosphate; FPP, farnesyl pyrophosphate; GGPP, geranylgeranyl pyrophosphate. Dashed arrows represent multi-step pathways; red X indicates the targeted deletion of the corresponding gene.

In this study, we selected and codon-optimized three β-carotene 15,15′-dioxygenases from the uncultured marine bacterium 66A03 (*Blh*), *Homo sapiens* (*BCDO*), and *Drosophila melanogaster* (*ninaB*), which were introduced into the genome of *R*. *toruloides* NP11. Retinal was first extracted by two-phase fermentation and detected by HPLC. Increasing *Blh* copy number and overexpressing the gene encoding isopentenyl-diphosphate isomerase *IDI1* were conducted to enhance the conversion of β-carotene to retinal. To further boost the flux of the precursor β-carotene, geranylgeranyl diphosphate synthase (*BTS1*) and hydroxymethylglutaryl-CoA reductase (*HMG1*) were also overexpressed. It was suggested that the intracellular concentration of β-carotene was elevated by 71.5% and 39.4%, respectively. Combinatorial exploration of a series of modifications was investigated, and varying degrees of product enhancement were found. We also studied different fermentation conditions such as temperature, antioxidants, extractants, and culture medium. It was observed that the highest titer of retinal reached 20.38 mg/L with fed-batch fermentation under the optimal conditions. Herein, the de novo biosynthesis of retinal in *R. toruloides* NP11 was successfully achieved, demonstrating the capability of this oleaginous yeast to synthesize terpenoids (Figure 1). This research provides both theoretical and empirical foundations for future metabolic engineering initiatives and the advancement of NP11 as a cell factory.

## 2. Materials and Methods

### 2.1. Strains and Medium

The strains and plasmids used in this study are listed in Table 1. The parental strain *R. toruloides* NP11 was obtained from Prof. Zongbao Zhao’s Lab [35]. All *R. toruloides* strains were grown at 30 °C with shaking at 220 rpm. The culture medium for *R. toruloides* strains was prepared as follows: YPD medium composed of 10 g/L yeast extract, 20 g/L peptone, and 20 g/L glucose was employed for cultivating and selecting *R. toruloides* strains, and SC medium containing 8 g/L SC complete medium and 20 g/L glucose was used for carotenoid and retinal production. The SC complete medium was purchased from LABLEAD (Beijing, China). The nitrogen-limited (NL) medium used for fermentation was composed of 50.0 g/L glucose, 1.5 g/L yeast extract, and 1.7 g/L yeast nitrogen base (YNB) without amino acids and ammonium sulfate (LABLEAD, Beijing, China). For yeast selection, antibiotics were used at the following concentrations: geneticin (G418) 200 μg/mL (Sangon Biotech, Shanghai, China), hygromycin (HYG) 50 μg/mL (Sangon Biotech, Shanghai, China), nourseothricin (NAT) 50 μg/mL (Maokangbio, Shanghai, China), and zeocin (BLE) 50 μg/mL (Maokangbio, Shanghai, China). *E. coli* DH5α (Sangon Biotech, Shanghai, China) cells harboring plasmid were cultured in Luria–Bertani (LB) medium supplemented with appropriate antibiotics (100 μg/mL ampicillin and 50 μg/mL kanamycin) for plasmid amplification at 37 °C for 16 h. Ampicillin and kanamycin were purchased from Sangon Biotech (Shanghai, China). To prepare solid medium, 2% (*w*/*v*) agar was added to the respective liquid LB and YPD media prior to sterilization.

### 2.2. Plasmid Construction

The β-carotene 15,15′-dioxygenase genes from the uncultured marine bacterium 66A03 (*Blh*), *H. sapiens* (*BCDO*), and *D. melanogaster* (*ninaB*) were codon-optimized and synthesized by Sangon Biotech (Shanghai, China) (Table S1). The three fragments along with the vector pZPK were digested with *Eco*RV/*Spe*I, followed by ligation using a DNA Ligation Kit (Takara, Beijing, China). The restriction enzymes were purchased from Takara (Beijing, China). Using *R. toruloides* genomic DNA as a template, the target genes and promoters were amplified for expression. Specifically, *BTS1*, *CARRP*, and *CARB* were linked via 2A peptides under the control of the *GPD* promoter; *HMG1* was driven by *LDP1* (both of which originated from the pZPK vector), and *ERG10*, *ERG13*, and *IDI1* were driven by endogenous promoters *RT14*, *FBA*, and *PGI*, respectively. All plasmids were constructed from pZPK using the Gibson assembly method [39]. The DNA gel purification kit, ClonExpress Ultra One Step Cloning Kit V2, and Phanta Max Super-Fidelity DNA Polymerase were sourced from Vazyme (Nanjing, China), while DNA and plasmid extraction kits were provided by TIANGEN (Beijing, China). The primers utilized in this investigation were synthesized by Sangon Biotech (Shanghai, China) and are detailed in Table S2. Guide RNA (gRNA) was designed via CRISPOR from Zhang’s lab (http://crispor.tefor.net/) [40]. To achieve *TGL* knockout, a plasmid was constructed to co-express Cas9 [38] and the gRNA (ACTCGCGCGACTGAGCTCTG) directed against the *TGL* (RHTO_00993) gene in *R*. *toruloides*, which was subsequently introduced into the NP11 strain via hygromycin selection. The genes employed in this study are listed in Table S3.

### 2.3. Yeasts Transformation and Screening

All plasmids were linearized with restriction endonuclease *Eco*RI (Takara) before transformation. These linearized fragments were then transformed into *R. toruloides* using the heat shock method as previously described [33]. However, for target fragments larger than 8 kb, the electroporation method was employed [41]. The transformants were spread onto selection YPD plates supplemented with the appropriate antibiotics and incubated at 30 °C for 3 days. To obtain correct transformants, genomic DNA was extracted using TIANamp Yeast DNA Kit (Beijing, China), followed by PCR amplification of the target locus for sequencing.

### 2.4. Shake Flasks Culture

To quantify the β-carotene levels in the recombinant strains, transformants were picked randomly and inoculated in 14-mL tubes with 3 mL YPD medium supplemented with appropriate antibiotics. Then, a fresh overnight culture was inoculated in 50 mL shake flasks containing 10 mL SC medium with an initial OD_600_ of 0.2. After 120 h cultivation, 1 mL of cell culture was taken for β-carotene extraction. For the retinal production, experiments were carried out in 150-mL flasks containing 20 mL SC medium supplemented with 20% dodecane, which were inoculated with fresh overnight cultures to an initial OD_600_ of 0.2. Dodecane was employed to capture retinal secreted externally during the fermentation process. According to the added dodecane, the retinal production was calculated after 84 h of cultivation. Dodecane was purchased from Aladdin (Shanghai, China).

### 2.5. Real-Time Quantitative PCR

The wild type (WT) strain was cultivated in 250-mL shake flasks with 50 mL SC medium and was sampled at 24 h and 72 h for transcription analysis. A 1 mL volume of cell culture was harvested and centrifuged at 13,000 rpm for 2 min, and the obtained cell pellet was then used for RNA extraction. Total RNA was extracted using the FastPure Cell/Tissue Total RNA Isolation Kit V2 (Vazyme, China), and the RNA concentration was determined using a MicroSpectrophotometer (KAIAO, Beijing, China). The reverse-transcribed cDNA was synthesized using HiScript^®^ III All-in-one RT SuperMix Perfect for qPCR (Vazyme, China). RT-qPCR was conducted on an Applied Biosystems QuantStudio 3^TM^ Real-Time PCR System (Thermo Fisher Scientific, Waltham, MA, USA) with Taq pro Universal SYBR qPCR Master Mix (Vazyme, China) and performed in triplicate. The native gene *ACTIN* was chosen as the reference gene for normalizing the expression levels [42]. The relative gene expression was calculated using the 2^−ΔΔCT^ method.

### 2.6. Analysis of Glucose, β-Carotene, Retinal

Glucose was determined by HPLC using a Shimadzu LC-20AT system (Shimadzu, Kyoto, Japan) equipped with a refractive index detector and an Aminex HPX-87H column (300 × 7.8 mm, Bio-Rad, Hercules, CA, USA). A 500 μL volume of culture was centrifuged at 13,000 rpm for 2 min, and the supernatant and mobile phase were diluted in a 1:1 ratio. The mobile phase consisted of 5 mM sulfuric acid with a flow rate of 0.6 mL/min at 40 °C.

For quantitation of β-carotene, 1 mL culture was centrifuged at 13,000 rpm for 2 min. Cell pellets were resuspended in 0.5 mL dimethyl sulfoxide (DMSO), and then incubated at 60 °C for 10 min until the cells were bleached. After addition of 1 mL acetone, the sample was briefly mixed and incubated at 50 °C for 15 min. The supernatant was analyzed by HPLC using a Shimadzu LC-20AT system with a UV detector (wavelength 450 nm) and equipped with a shim-pack Scepter C18 column (5 μm, 4.6 × 250 mm). The mobile phase to detect β-carotene consisted of acetonitrile–methanol–isopropanol (5:3:2 *v*/*v*) and was run with a flow rate of 1 mL/min at 40 °C.

In the two-phase culture with dodecane, the retinal concentration was calculated based on the volume of the dodecane layer. To determine retinal, all the culture was collected and centrifuged for 15 min at 10,000 rpm at 4 °C. The upper dodecane phase was analyzed by HPLC using the same system as β-carotene, except that the wavelength was 370 nm and the mobile phase consisted of acetonitrile–methanol (95:5 *v*/*v*). Standards of retinal and β-carotene were obtained from Acmec (Shanghai, China).

### 2.7. Fed-Batch Fermentation

For fed-batch fermentation, a single colony was selected and incubated in 14-mL tubes with 3 mL YPD medium for 16 h to obtain the primary seed, and the secondary seed was prepared by adding 0.5 mL primary seed into a 250-mL shake flask containing 50 mL of fresh YPD medium for 24 h (30 °C, 220 rpm). Then seed cultures were transferred to a 1.5-L fermenter (CloudReady^TM^, Parallel-Bioreactor, Shanghai, China) containing 500 mL SC medium with an initial inoculation volume of 5% (*v*/*v*). Antibiotics were added to the fermentation medium if required. The temperature was kept at 30 °C, and the pH was automatically adjusted to 5.6 using 1 M sodium hydroxide or 1 M citric acid. Agitation speed was set between 200 and 600 rpm to ensure dissolved oxygen levels remained above 30% during fermentation, with an aeration rate of 2.0 vvm. When the residual glucose content fell below 5 g/L, glucose was added to maintain a concentration of about 30 g/L until fermentation concluded. Samples were collected every 12 h to analyze residual glucose, dry cell weight, and retinal.

## 3. Results

### 3.1. Cultivation Medium Influences β-Carotene Production

**Figure 2:**
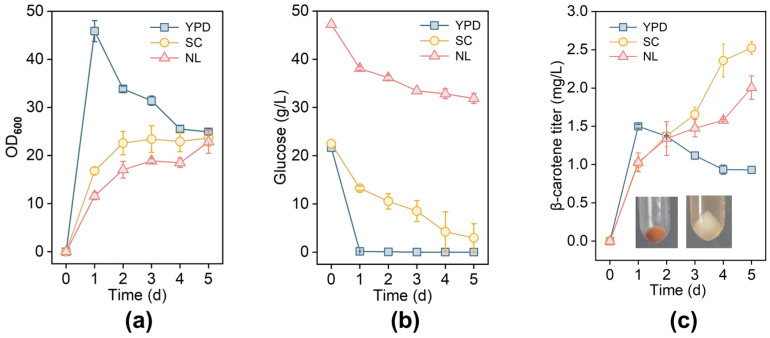
β-carotene synthesis in different media by NP11. The OD_600_ (cell growth) (**a**) and glucose consumption (**b**) of NP11 in different media. (**c**) Production of β-carotene in different media. Inset: Color change before and after extraction.

*R. toruloides* naturally produces carotenoids, including γ-carotene, β-carotene, torularhodin, and torulene. The culture medium significantly affects the product ratios; for instance, the β-carotene content varies with the C/N ratio [43]. To determine the optimal fermentation medium, we monitored the growth curves and glucose consumption of NP11 in YPD, SC complete medium, and NL medium. As shown in Figure 1, the strains exhibited the best growth and glucose consumption levels in YPD medium (Figure 2a,b), but the highest β-carotene production (2.52 mg/L) occurred in SC medium (Figure 2c). Additionally, nitrogen-limited conditions promote lipid production through competitive pathways, reducing carbon flow to β-carotene synthesis [29,44]. Consequently, SC medium was chosen for further fermentation studies.

### 3.2. Construction of Retinal-Producing R. toruloides Strains

**Figure 3:**
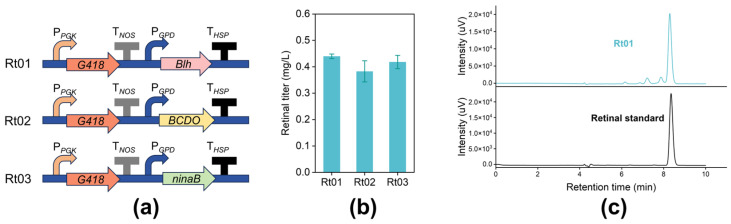
Selection of BCOs for synthesis of retinal in NP11. (**a**) Schematic diagram of the expression cassettes with *Blh*, *BCDO* and *ninaB* driven by *GPD* promoter. P*_PGK_*, phosphoglycerate kinase promoter; *G418*, geneticin resistance gene; P*_GPD_*, glyceraldehyde-3-phosphate dehydrogenase promoter; T*_NOS_* and T*_HSP_* represent the *NOS* and *HSP* terminators, respectively. (**b**) Retinal production by the recombinant strains. (**c**) HPLC profile of a retinal standard and the upper layer of Rt01 fermentation medium.

Since *R. toruloides* NP11 possesses the native complete MVA pathway and carotenoid synthesis pathway, we only need to introduce BCO to convert β-carotene into retinal. We screened BCOs from three species previously shown to effectively transform β-carotene to retinal in vitro: *Blh* from an uncultured marine bacterium 66A03, *BCDO* from *Homo sapiens*, and *ninaB* from *Drosophila melanogaster* [45]. After codon optimization, these BCOs were expressed under the glyceraldehyde-3-phosphate dehydrogenase promoter (P*_GPD_*) and then transferred to NP11 to assess their retinal synthesis capabilities (Figure 3a). Given that retinal is hydrophobic and chemically unstable, we established a two-phase culture system by adding an extractant into the fermentation medium to facilitate product secretion [46]. The organic phase from the upper fermentation layer was collected for product content analysis. Among the engineered candidates, the strain Rt01 expressing the codon-optimized *blh* gene from the uncultured marine bacterium 66A03 achieved a retinal titer of 0.44 mg/L (Figure 3b,c). These results indicate that the optimized 66A03-derived BCO construct possesses enhanced in vivo catalytic capacity for retinal production in *R. toruloides* compared to the other tested variants, likely due to improved protein expression within this host.

### 3.3. Enhancing β-Carotene Production to Boost Retinal Precursor Supply

**Figure 4:**
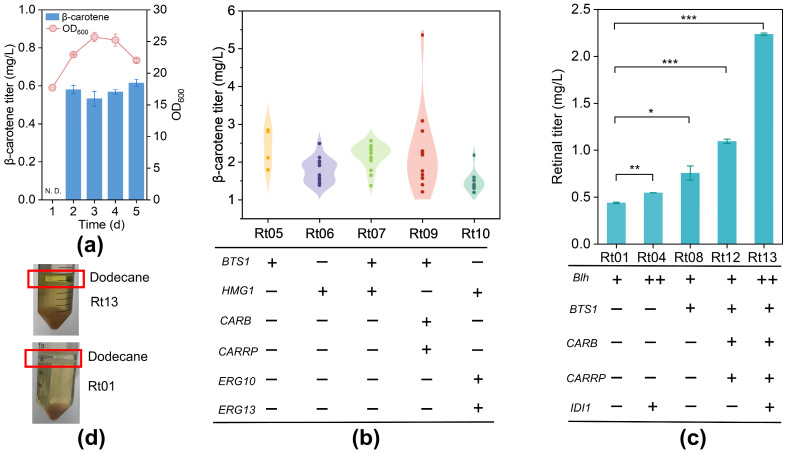
Improving retinal synthesis by enhancing precursor availability. (**a**) Cell growth (OD_600_) and β-carotene accumulation changes in the Rt01 strain during 5 days of fermentation. (**b**) Comparison of β-carotene production among various recombinant strains, with Rt05, Rt06, and Rt07 sampled at 60 h, and Rt09 and Rt10 at 120 h. (**c**) A synergistic approach to metabolic engineering strategies led to increased retinal production. (**d**) Differences observed in the dodecane layer between Rt13 and Rt01 fermentation cultures. Statistical significance was assessed using Student’s *t* test (*, *p* < 0.05; **, *p* < 0.01; ***, *p* < 0.001). N.D., not detected.

The intracellular concentration of β-carotene in strain Rt01 was monitored over 5 days of fermentation in SC medium. No β-carotene was detected on the first day, but its subsequent accumulation over the following four days suggests that the rate of β-carotene consumption by BCO was insufficient to balance the upstream biosynthetic flux during this period (Figure 4a). To improve β-carotene conversion, we increased the copy number of *Blh* to elevate BCO levels. In addition, we constructed a plasmid containing a cassette with *IDI1*, which encodes isopentenyl-diphosphate isomerase, along with two *Blh* expression cassettes. Enhancing the expression of IDI1, a key rate-limiting enzyme, is crucial for increasing the metabolic flux in the MVA pathway [47]. Strain Rt04, harboring two copies of the *blh* gene and overexpressing the *IDI1* gene, exhibited a 25% increase in retinal production compared to Rt01 (Figure 4c). Although increasing the *Blh* copy number proved effective for the metabolic conversion of β-carotene to retinal, the still-limited retinal production indicated a need for more precursor supply. Therefore, addressing the rate-limiting steps in the carotenoid synthesis pathway is essential for increasing β-carotene availability.

BTS1 (geranylgeranyl diphosphate synthase) and HMG1 (hydroxymethylglutaryl-CoA reductase), which catalyze the conversion of geranylgeranyl pyrophosphate (GGPP) to phytoene and the conversion of hydroxymethylglutaryl-CoA to mevalonate, respectively, are two critical rate-limiting enzymes in the carotenoid biosynthesis pathway [48,49]. We evaluated the effects of overexpressing *BTS1* and *HMG1* in strain NP11 by constructing strains Rt05 and Rt06 with endogenous BTS1 and HMG1, respectively, and strain Rt07, which features both enzymes driven by strong promoters. The β-carotene levels in Rt05, Rt06, and Rt07 were 71.5%, 39.4%, and 51.5% higher, respectively, compared to that in the WT strain NP11 (Figure 4b). We constructed strain Rt08 by co-expressing the *BTS1* and *blh* genes, which exhibited a 72.7% increase in retinal production compared to Rt01 (Figure 4c). This notable enhancement in retinal yield underscores the importance of optimizing both precursor supply and key gene expression for maximizing retinal production.

We also implemented two additional strategies to enhance precursor supply: boosting the carotenoid biosynthesis and MVA pathway. We created strain Rt09 by placing *CARRP* (encoding phytoene synthase), *CARB* (encoding phytoene dehydrogenase), and BTS1 in a single expression cassette using 2A peptides, a method previously established in *R*. *toruloides* [50,51]. Simultaneously, we overexpressed *ERG10* (encoding acetoacetyl-CoA thiolase), *ERG13* (encoding hydroxymethylglutaryl-CoA synthase), and *HMG1* by cloning them into three separate cassettes to form strain Rt10. Strain Rt09 achieved the highest β-carotene production, reaching 2.3 times that of NP11 (Figure 4b), while Rt10 did not enhance β-carotene levels. We then integrated *Blh* expression cassette or dual *Blh* expression cassettes with *IDI1* into Rt09 (which already contains the carotenoid module) to generate Rt12 and Rt13, respectively, enabling this strong β-carotene producer to generate retinal. Strain Rt13 yielded 2.24 mg/L of retinal, which is 5.1 times higher than that of Rt01, with a concomitantly deeper color in the upper dodecane layer (Figure 4d).

Due to the random integration of heterologous genes into the *R. toruloides* genome, significant production variation was observed among the initial transformants (Figure 4b). This variability, likely arising from chromosomal position effects, necessitated a comprehensive preliminary screening of a large number of colonies to identify candidates with a high capacity for β-carotene accumulation. Utilizing a similar screening strategy, we systematically screened the retinal-producing transformants to identify those with the optimal retinal titers. The subsequent stability observed in Figure 4c reflects the consistent performance of the selected top-performing transformants during fermentation.

### 3.4. Disrupting the Triacylglyceride Oxidation Pathway to Improve the Availability of Lipid-Soluble Carriers

**Figure 5:**
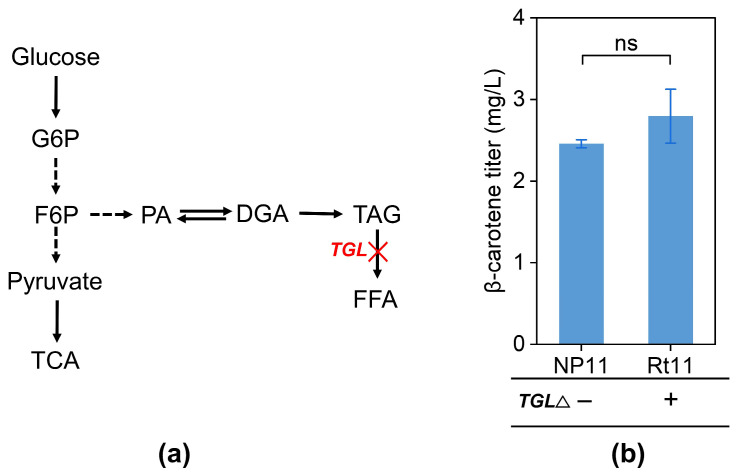
Knockout of *TGL*. (**a**) Pathway of triacylglycerol (TAG) synthesis in *R. toruloides* NP11. G6P, glucose-6-phosphate; F6P, fructose-6-phosphate; PA, phosphatidic acid; DAG, diacylglycerol; TAG, triacylglycerol; FFA, free fatty acid; TGL, triacylglycerol lipase; TCA, tricarboxylic acid cycle. (**b**) Results of fermentation by the WT and knockout strains. Statistical significance was assessed using Student’s *t*-test. (ns, *p* > 0.05). Solid arrows indicate single-step enzymatic reactions; dashed arrows represent multi-step pathways; red X indicates the targeted deletion of the corresponding gene.

Triacylglycerides (TAGs) serve as intracellular carriers for lipid-soluble terpenoids, facilitating their transport and utilization within cells [52,53]. Inactivating the *TGL* gene family is thought to significantly boost lipid accumulation, particularly TAG, thereby enhancing the cell’s capacity to store lipid-soluble compounds (Figure 5a). To explore this, a CRISPR/Cas9 deletion system [38] was implemented in *R*. *toruloides* to create the mutant strain Rt11 with an inactive *TGL* gene. However, unexpectedly, the lack of TGL only resulted in a modest 13.7% increase in intracellular β-carotene levels (Figure 5b). Owing to the reduced transformation efficiency associated with the large CRISPR-Cas9 cassette, the implementation of complex genetic manipulations such as fusing Cas9 with transcriptional effectors remains a significant challenge. Consequently, this study focused on evaluating the effects of gene deletions on β-carotene production without the exogenous insertion of additional DNA fragments.

### 3.5. Optimization of Culture Conditions for Retinal Production

**Figure 6:**
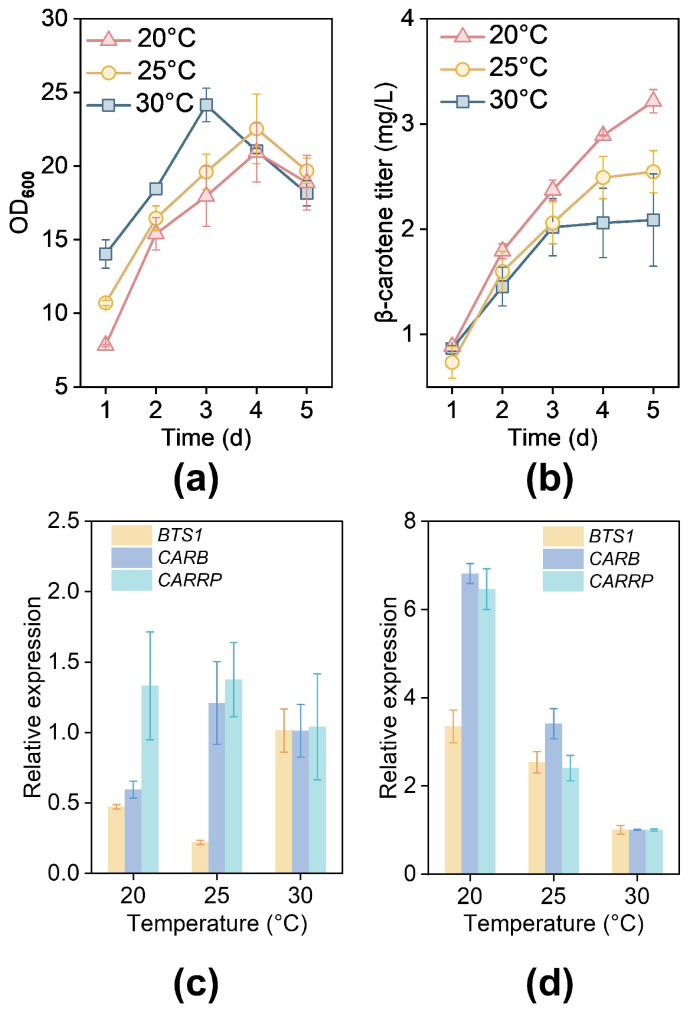
Temperature optimization for cultivation in SC medium. (**a**) Growth performance of NP11 at 20 °C, 25 °C, and 30 °C. (**b**) β-carotene accumulation by NP11 at different temperatures. Transcriptional levels of genes associated with the β-carotene biosynthetic pathway under temperatures of 20 °C and 25 °C at 24 h (**c**) and 72 h (**d**).

To improve retinal production in *R. toruloides*, we sequentially optimized three factors: temperature, antioxidants, and extractants. Recognizing that maximizing precursor availability is a prerequisite for efficient product conversion, we systematically evaluated β-carotene accumulation at 20 °C, 25 °C, and 30 °C. This initial optimization was essential to establish a solid metabolic foundation for the subsequent retinal biosynthesis stage. As illustrated in Figure 6a,b, growth was slightly inhibited at 20 °C and 25 °C compared to that at 30 °C. Additionally, after 5 days of cultivation, we observed that higher temperatures resulted in decreased β-carotene biosynthesis. After 72 h of cultivation, the expression levels of the endogenous genes *BTS1*, *CARB*, and *CARRP*, involved in carotenoid biosynthesis, were significantly higher at 20 °C than at 30 °C (Figure 6c,d), with up-regulations of 3.35-fold for *BTS1*, 6.81-fold for *CARB*, and 6.46-fold for *CARRP*. Thus, lower temperatures favor β-carotene accumulation.

**Figure 7:**
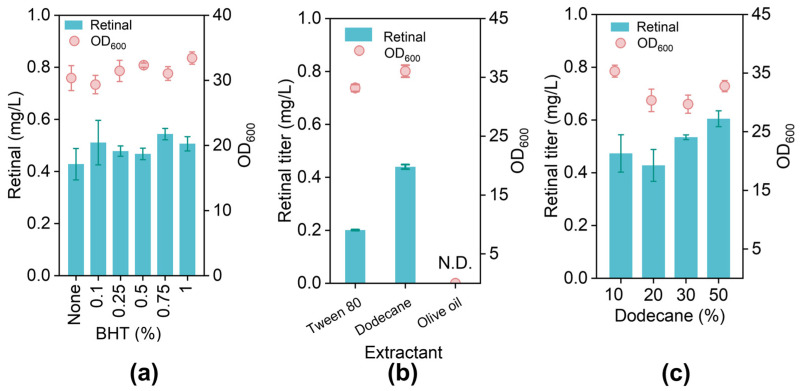
Optimization of the antioxidant (BHT) and extractants. (**a**) Retinal production induced by adding various concentrations of BHT. (**b**) Comparison of different solvent extraction efficiencies. (**c**) Optimization of the dodecane volume ratio. All optimization experiments were conducted using the engineered strain Rt01. N.D., not detected.

To mitigate oxidative degradation of carotenoids during production and storage, we incorporated antioxidants [54]. Here, butylated hydroxytoluene (BHT) was utilized as a stabilizer, and we examined the effects of varying its ratio on retinal yield. Notably, different BHT ratios had minimal impact on retinal production and did not significantly affect cell growth (Figure 7a). Next, we aimed to establish a two-phase culture system conducive to retinal synthesis by comparing the in situ extraction efficiency of three commonly used extractants: Tween 80, dodecane, and olive oil. The results indicated that the retinal yield with dodecane was double that of Tween 80, while olive oil significantly inhibited yeast growth (Figure 7b). Therefore, dodecane was chosen as the optimal retinal extractant. We also assessed the effect of extractant volume, finding that the best extraction occurred with 50% (*v*/*v*) dodecane in the fermentation broth (Figure 7c).

Under these optimized conditions, the previously engineered strain Rt13 was fermented, achieving a retinal production of 3.2 mg/L, which is 7.3 times higher than that of strain Rt01. These results demonstrate that the systematic metabolic engineering strategy, combined with the optimization of fermentation conditions, significantly enhanced retinal accumulation in the engineered strains.

### 3.6. Fed-Batch Fermentation of Retinal in a 1.5 L Bioreactor

**Figure 8:**
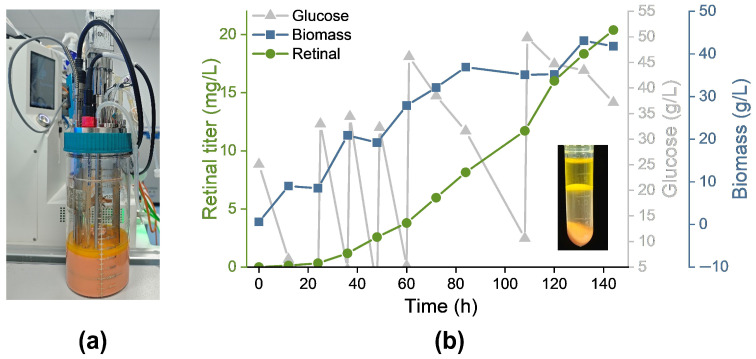
Production of retinal by the Rt13 strain via fed-batch fermentation in a 1.5-L bioreactor. (**a**) The appearance of the Rt13 strain following 144 h of cultivation in the bioreactor. (**b**) Time course of biomass accumulation, glucose utilization, and retinal synthesis by the Rt13 strain during the fed-batch process. Inset: a tube containing retinal extracted into dodecane.

To assess the ability of *R. toruloides* Rt13 to produce retinal in a high cell-density environment, a 144-h fermentation was performed in a 1.5-L bioreactor (Figure 8a). Key parameters, including temperature, pH, dissolved oxygen, and agitation speed, were monitored throughout the process. An extractant, 100 mL of dodecane, was added, and samples were taken at regular intervals to evaluate biomass and retinal production. This allowed for a thorough analysis of productivity and yield during fermentation. The findings highlighted the metabolic performance of Rt13 in a high-density culture. At the end of fermentation, the dodecane layer displayed a noticeable yellow hue, with a retinal concentration of 20.38 mg/L (Figure 8b). Retinal production in the bioreactor significantly surpassed that from shake flask cultivation.

Retinal production was normalized to biomass (mg/g DCW) to evaluate the biosynthetic efficiency across different cultivation scales. In shake flasks, strain Rt13 achieved a specific yield of 0.45 mg/g DCW. Upon scaling up to a 1.5 L bioreactor, although the biomass and total titer increased significantly to 41.8 g/L and 20.38 mg/L, respectively, the specific yield remained comparable at 0.49 mg/g DCW. This comparable per-cell efficiency during high-density fermentation suggests significant potential for further systematic optimization of the bioreactor process. Future strategies to enhance specific yield include refining oxygen transfer rates to maintain optimal dissolved oxygen, implementing precise nutrient feeding to balance primary metabolism with heterologous flux, and conducting a more comprehensive optimization of medium constituents. Such adjustments will better align metabolic driving forces with increased cell density, potentially leading to substantial improvements in both specific yield and final product concentration.

## 4. Discussion

*R*. *toruloides* was selected as the expression host due to its robust metabolic pathways that efficiently channel carbon flux into acetyl-CoA and malonyl-CoA, providing a consistent supply of precursors for isoprenoid biosynthesis [33]. The native mevalonate (MVA) pathway and innate carotenogenic background of *R. toruloides* create a highly compatible metabolic environment for the derivation of carotenoids [55]. Furthermore, the ability of *R. toruloides* to accumulate lipids reaching over 60% of its cell dry weight under certain conditions results in the formation of lipid bodies that provide an ideal physical sanctuary for the efficient production and stable storage of hydrophobic carotenoids [25].

In this study, we screened BCOs from various species and introduced them into *R*. *toruloides* to create retinal-producing strains. Retinal is lipophilic, so we added extractants to establish a two-phase culture system to improve product recovery from the fermentation medium [56]. The expression level and catalytic capacity of BCO, as the key enzyme for retinal synthesis, are critical factors in maximizing production. Consequently, many studies have focused on increasing the copy number of *Blh* to improve the production of retinoids [49,57,58]. For instance, 11 copies of *Blh* were integrated into the genome of a β-carotene-producing yeast strain, achieving high retinol levels during fed-batch fermentation [49]. Strain Rt04 was constructed by increasing the copy number of *Blh* to enhance the yield of retinal. It was suggested that the enhancement of BCO may improve the metabolic flux from β-carotene to retinal. However, the limited retinal production may be due to an insufficient supply of precursors, hindering the full potential of BCO activity.

It is hypothesized that carotenoid production can be improved by enhancing the MVA pathway or channeling acetyl-CoA towards carotenogenesis, by either upregulating the MVA pathway or downregulating lipid biosynthesis [55]. Thus, we employed a modular expression approach to independently introduce the carotenoid synthesis module and the MVA synthesis module into *R. toruloides* to generate strains Rt09 and Rt10, respectively. The carotenoid module contained *CARB*, *BTS1*, and *CARRP*, while the MVA module comprised *ERG10*, *ERG13*, and *HMG1*. The results indicated that Rt09 achieved the highest β-carotene production, representing a 112.7% increase compared to that in NP11. At the same time, Rt10 showed no significant difference in β-carotene production, implying that enhancing the MVA pathway may not be essential. Previous studies have shown that overexpressing endogenous or exogenous *CARRP* and *CARB* in various host organisms, like *S. cerevisiae* and *Y. lipolytica*, can lead to a high carotenoid production [59,60,61]. Therefore, strengthening the carotenoid synthesis module is a more feasible method than boosting the MVA module for enhancing β-carotene production.

Carotenoid accumulation is closely linked to fatty acid production, as liposomes act as reservoirs for lipid-soluble carotenoids. Studies have indicated a positive relationship between lipid accumulation and carotenoid production in *R. toruloides* [62]. However, deleting the triacylglycerol lipase gene *TGL* resulted in only a slight increase in β-carotene production. This complex interaction between carotenoids and lipid accumulation in *R*. *toruloides*, involving a competition for acetyl-CoA and mutual stimulation of biosynthesis, suggests that targeting only one pathway may not effectively enhance β-carotene accumulation [55].

Environmental factors frequently interfere with cellular metabolism during industrial fermentation, affecting titer, yield, and productivity. Therefore, we optimized temperature, antioxidants, and extractants sequentially. Lower temperatures enhance cellular metabolism regulation and increase the expression of ribosome-related genes, promoting protein biosynthesis [63]. A previous study indicated that β-carotene accumulation rose as the temperature decreased, likely due to a reduced flux through the TCA cycle, resulting in more carbon directed into the MVA pathway and stimulating terpenoid production [64]. Similarly, tocotrienols were produced efficiently during a two-stage fermentation process with a low-temperature control system [65]. In *S. cerevisiae*, taxadiene titers increased 1.3-fold at 20 °C compared to 30 °C [66]. BHT is widely used as an antioxidant in food, cosmetics, and pharmaceuticals to prolong shelf life [67]. Numerous studies have demonstrated that BHT treatment effectively prevents the degradation of retinol produced by *S. cerevisiae* [68]. Research has also explored various antioxidants to enhance yields in vitamin A biosynthesis by microorganisms. Adding BHT to different vitamin A-producing microorganisms boosted total vitamin A production [49,69]. Adjusting cellular metabolism via environmental conditions is a more adaptable and straightforward method compared to the complexities of genetic modifications [64,70,71].

In this study, we achieved a retinal production that demonstrates the potential of *R. toruloides* as a robust microbial cell factory. However, a comparison with established benchmarks in other yeast models reveals considerable room for advancement. For instance, multidimensional engineering of *S. cerevisiae* has achieved a record retinol titer of 7.19 g/L in a 5 L bioreactor, with a yield of 0.0106 g/g glucose and a productivity of 0.055 g/L·h [72]. Similarly, engineered *Y. lipolytica* has reached a titer of 4.86 g/L, boasting a superior yield of 0.016 g/g glucose and a productivity of 0.047 g/L·h [49].

Although *R. toruloides* currently trails established industrial models in retinal titers, its vast acetyl-CoA flux offers a superior metabolic foundation for terpenoid biosynthesis. To bridge this gap, future efforts must address several key bottlenecks. Primarily, enhancing heterologous gene expression through the identification of stable integration sites or high-copy-number plasmids is essential. Furthermore, protein engineering to refine enzymatic specificity and subcellular compartmentalization (e.g., lipid body sequestration) can redirect metabolic flux toward β-carotene while mitigating the cellular toxicity of lipophilic intermediates. To overcome the stochasticity of non-homologous end joining (NHEJ)-mediated integration, high-throughput screening will be instrumental in identifying rare, high-yielding variants. Additionally, modulating membrane transporters may alleviate the intracellular retention of hydrophobic retinoids [73,74]. By synergizing these genetic and bioprocess engineering strategies, *R. toruloides* is poised to become a highly efficient and robust platform for high-value carotenoid production.

## 5. Conclusions

This study explored retinal production in *R. toruloides*, a non-conventional yeast known for its ability to accumulate lipids and synthesize carotenoids. By introducing β-carotene dioxygenases from various sources, we developed retinal-producing strains, with *Blh* exhibiting the highest conversion efficiency. A two-phase culture system improved the secretion of carotenoids and retinoids, facilitating product recovery. Our results indicated that nitrogen-limiting conditions in the SC medium boosted retinal production, likely by minimizing by-product formation compared to YPD medium. Furthermore, increasing the copy number of *Blh* and overexpressing key genes such as *BTS1* and *HMG1* in the MVA pathway enhanced yields of β-carotene and retinal, highlighting the significance of precursor availability in carotenoid synthesis.

However, competition between lipid and carotenoid biosynthesis for acetyl-CoA posed challenges for optimizing production. Although improving the carotenoid synthesis pathway was beneficial, further improvements might necessitate adjustments in metabolic flux and membrane transport to enhance the secretion of lipid-soluble compounds like retinal. Environmental factors, such as temperature and antioxidants, were also crucial, with lower temperatures favoring terpenoid synthesis and β-carotene accumulation.

In summary, *R. toruloides* has significant potential for sustainable retinoid production through metabolic engineering. Future research should focus on optimizing metabolic pathways, refining extraction methods, and modulating cell membrane characteristics to maximize yields. These findings contribute to the field of retinoid biosynthesis and lay the groundwork for industrial-scale production.

## Figures and Tables

**Table 1:** Plasmids and strains used in this study.

Plasmids/Strains	Description	Source
Plasmids		
pZPK	*Agrobacterium*-mediated integration vector, *Kan*^R^	[36]
pZPK-P*_PGK_*-*HYG^R^*-T*_NOS_*-P*_GPD_*-MCS-T*_HSP_*	P*_PGK_*-*HYG^R^*-T*_NOS_* in pZPK, *Kan*^R^	[36]
pZPK-P*_PGK_*-BLE*^R^*-T*_NOS_*-P*_GPD_*-MCS-T*_HSP_*	P*_PGK_*-*BLE^R^*-T*_NOS_* in pZPK, *Kan*^R^	[37]
pZPK-P*_PGK_*-G418*^R^*-T*_NOS_*-P*_GPD_*-MCS-T*_HSP_*	P*_PGK_*-*G418^R^*-T*_NOS_* in pZPK, *Kan*^R^	[37]
pZPK-P*_PGK_*-NAT*^R^*-T*_NOS_*-P*_LDP_*-MCS-T*_HSP_*	P*_PGK_*-*NAT^R^*-T*_NOS_* in pZPK, *Kan*^R^	[37]
NM8-5S-tRNA-tRNA-SgH	gRNA Helper Plasmid, P*_GPD1_*-*HYG^R^*-T*_NC_* in pCambiao380, *Kan*^R^	[38]
Strains		
*Rhodosporidium toruloides* NP11	Haploid, MAT A	[35]
NP11-*SpCas9*	NP11, pZPK-P*_PGK_*-*G418^R^*-T*_NOS_*-P*_PGK_*-*SpCas9*-NLS3-T*_35S_*	[38]
Rt01	NP11/pRt01, pZPK-P*_PGK_*-*G418^R^*-T*_NOS_*-P*_GPD_*-*Blh*-T*_HSP_*	This study
Rt02	NP11/pRt02, pZPK-P*_PGK_*-*G418^R^*-T*_NOS_*-P*_GPD_*-*BCDO*-T*_HSP_*	This study
Rt03	NP11/pRt03, pZPK-P*_PGK_*-*G418^R^*-T*_NOS_*-P*_GPD_*-*ninaB*-T*_HSP_*	This study
Rt04	NP11/pRt04, pZPK-P*_PGK_*-*G418^R^*-T*_NOS_*-P*_GPD_*-*Blh*-T*_HSP_*-P*_LDP1_*-*Blh*-T*_HSP_*-P*_PGI_*-*IDI1*-T*_HSP_*	This study
Rt05	NP11/pRt05, pZPK-P*_PGK_*-*NAT^R^*-T*_NOS_*-P*_LDP1_*-*BTS1*-T*_HSP_*	This study
Rt06	NP11/pRt06, pZPK-P*_PGK_*-*NAT^R^*-T*_NOS_*-P*_LDP1_*-*HMG1*-T*_HSP_*	This study
Rt07	NP11/pRt07, pZPK-P*_PGK_*-*NAT^R^*-T*_NOS_*-P*_RT32_*-*BTS1*-T*_HSP_*-P*_LDP1_*-*HMG1*-T*_HSP_*	This study
Rt08	Rt05/pRt08, pZPK-P*_PGK_*-*G418^R^*-T*_NOS_*-P*_GPD_*-*Blh*-T*_HSP_*	This study
Rt09	NP11/pRt09, pZPK-P*_PGK_*-*BLE^R^*-T*_NOS_*-P*_GPD_*-*BTS1*-P2A-*CARB*-P2A-*CARRP*-T*_HSP_*	This study
Rt10	NP11/pRt10, pZPK-P*_PGK_*-*NAT^R^*-T*_NOS_*-P*_RT14_*-*ERG10*-T*_HSP_*-P*_FBA_*-*ERG13*-T*_HSP_*-P*_LDP1_*-*HMG1*-T*_HSP_*	This study
Rt11	NP11/pRt11, pZPK-P*_GPD1_*-*HYG^R^*-T*_NC_*-P*_5S-tRNA_*-g*TGL*-P*_PGK_*-*SpCas9*-NLS3-T*_35S_*	This study
Rt12	Rt09/pRt12, pZPK-P*_PGK_*-*G418^R^*-T*_NOS_*-P*_GPD_*-*Blh*-T*_HSP_*	This study
Rt13	Rt09/pRt13, pZPK-P*_PGK_*-*G418^R^*-T*_NOS_*-P*_GPD_*-*Blh*-T*_HSP_*-P*_LDP1_*-*Blh*-T*_HSP_*-P*_PGI_*-*IDI1*-T*_HSP_*	This study

Notes: P*_GPD1_*, glyceraldehyde-3-phosphate dehydrogenase promoter; P*_LDP1_*, lipid droplet protein 1 promoter; P*_PGK_*, phosphoglycerate kinase promoter; P*_PGI_*, glucose-6-phosphate isomerase promoter; P*_FBA_*, fructose 1,6-biphosphate aldolase promoter; P*_RT14_*, elongation factor EF-1 alpha subunit promoter; P*_RT32_*, glyceraldehyde-3-phosphate dehydrogenase promoter. T*_NOS_*, T*_35S_*, and T*_HSP_* represent the *NOS*, *35S*, and *HSP* terminators, respectively. *HYG*, hygromycin B resistance gene; *BLE*, bleomycin resistance gene; *G418*, geneticin resistance gene; *NAT*, nourseothricin resistance gene. P*_GPD1_*, P*_PGK_*, and P*_LDP1_* are promoters derived from the pZPK vector; P*_RT32_*, P*_RT14_*, P*_FBA_*, and P*_PGI_* are endogenous promoters amplified from the *R. toruloides* genomic DNA.

## Data Availability

The original contributions presented in this study are included in the article/Supplementary Materials. Further inquiries can be directed to the corresponding authors.

## Supplementary Materials

The following supporting information can be downloaded at: https://www.mdpi.com/article/10.3390/jof12040258/s1, Table S1: DNA sequences of the exogenous genes expressed in *R. toruloides*. Table S2: Primers used in this study. Table S3: The genes used in this study.
