# Supplementary material for: Metabolic Engineering of Rhodotorula toruloides for Biosynthesis of Retinal
Source: J Fungi (Basel). 2026 Apr 2;12(4):258. doi: 10.3390/jof12040258 (PMC13118192; doi:10.3390/jof12040258)
Supplement: Supplementary file 1 [file jof-12-00258-s001.zip › jof-4203805-supplementary.pdf]

Supplementary Information

Metabolic engineering of *Rhodotorula toruloides* for biosynthesis of retinal

Huihui Qiu [1,†], Linyue Tian [1,2,†], Lin Hu [1], Lianwu Wu [1], Yu Huang [1], Ran Ge [1], Yufan Xing [1],
Alexander A. Kamnev [3], Ning He [1,*] and Mingfeng Cao [1,2,*]

[1] Department of Chemical and Biochemical Engineering, College of Chemistry and Chemical Engineering, Key Laboratory for Synthetic Biotechnology of Xiamen City, Xiamen University, Xiamen 361005, China; 20620240156801@stu.xmu.edu.cn (H.Q.); tly3110570@163.com (L.T.); hulin@stu.xmu.edu.cn (L.H.); lianwuwu@stu.xmu.edu.cn (L.W.); yuhuang_0012@163.com (Y.H.); geran@stu.xmu.edu.cn (R.G.); yufan18888@163.com (Y.X.)
[2] Innovation Laboratory for Sciences and Technologies of Energy Materials of Fujian Province (IKKEM), Xiamen 361005, China
[3] Institute of Biochemistry and Physiology of Plants and Microorganisms, Saratov Federal Scientific Center of the Russian Academy of Sciences, Saratov 410049, Russia; a.a.kamnev@mail.ru
* Correspondence: hening@xmu.edu.cn (N.H.); mfcao@xmu.edu.cn (M.C.)
† These authors contributed equally to this work.

Table S1: DNA sequences of the exogenous genes expressed in *R. toruloides*.

**Genes**	**Sequences (5'-3')**
Blh	ATGGGCCTCATGCTGATCGACTGGTGCGCGCTCGCCCTCGTCGTGT<br>TCATCGGCCTCCCGCACGGCGCCCTCGACGCCGCCATCTCGTTCTC<br>GATGATCTCGTCCGCCAAGCGGATCGCCCGCCTCGCCGGCATCCTC<br>CTCATCTACCTCCTCCTCGCGACCGCGTTCTTCCTCATCTGGTACCA<br>GCTCCCGGCCTTCAGCCTCCTCATCTTCCTCCTCATCAGCATCATCC<br>ACTTCGGCATGGCCGACTTCAACGCCTCGCCCTCGAAGCTCAAGTG<br>GCCCCACATCATCGCCCACGGCGGCGTCGTCACCGTCTGGCTCCCT<br>CTCATCCAGAAGAACGAGGTCACCAAGCTCTTCTCGATCCTCACGA<br>ACGGCCCGACGCCGATCCTCTGGGACATCCTCCTCATCTTCTTCCTC<br>TGCTGGTCCATCGGAGTCTGCCTCCACACGTATGAGACCCTCCGCT<br>CGAAGCACTACAACATCGCGTTCGAGCTCATCGGCCTCATCTTCCT<br>CGCCTGGTACGCGCCGCCGCTCGTCACCTTCGCCACCTATTTCTGCT<br>TCATCCACTCCCGCCGCCACTTCTCCTTCGTCTGGAAGCAGCTCCAG<br>CACATGTCGTCGAAGAAGATGATGATCGGCTCGGCCATCATCCTCT<br>CGTGCACCTCGTGGCTCATCGGCGGCGGCATCTACTTCTTCCTCAA<br>CTCGAAGATGATCGCCTCGGAAGCCGCGCTCCAGACCGTCTTTATC<br>GGCCTCGCAGCGTTGACGGTCCCGCACATGATCCTCATCGACTTCA<br>TCTTCCGCCCCCACTCGTCGCGCATCAAGATTAAGAACTAGACTAG<br>T

BCDO	ATGGACATCATCTTCGGCCGCAACCGCAAGGAGCAGCTCGAGCCG<br>GTCCGCGCCAAGGTCACCGGCAAGATCCCTGCGTGGCTCCAGGGC<br>ACGCTCCTCCGCAACGGCCCTGGCATGCACACGGTCGGAGAGTCGC<br>GCTACAACCACTGGTTCGACGGGCTCGCGCTCCTCCACTCGTTCAC<br>CATCCGCGACGGCGAGGTCTACTACCGCTCGAAGTACCTCCGCTCG<br>GACACGTACAACACGAACATCGAGGCCAACCGCATCGTCGTGAGC<br>GAGTTCGGCACGATGGCGTACCCTGACCCATGCAAGAACATCTTCT<br>CCAAGGCGTTCTCGTACCTCTCGCACACCATCCCGGACTTCACCGA<br>CAACTGCTTGATCAACATCATGAAGTGCGGCGAGGACTTCTACGCC<br>ACCTCGGAGACCAACTACATCCGCAAGATCAACCCACAGACCCTC<br>GAGACCCTCGAGAAGGTCGACTACCGCAAGTACGTCGCCGTCAAC<br>TTGGCCACCTCGCACCCCCACTACGACGAGGCCGGCAACGTCCTCA<br>ACATGGGCACGTCGATCGTTGAGAAGGGCAAGACCAAGTACGTCA<br>TCTTCAAGATCCCCGCCACCGTCCCGGAGGGCAAGAAGCAGGGCA<br>AGTCGCCGTGGAAGCACACCGAGGTCTTTTGCTCGATCCCGTCCCG<br>ATCCCTCCTCTCGCCGTCGTACTACCACTCGTTCGGCGTCACCGAG<br>AACTACGTCATCTTCCTCGAGCAGCCGTTCCGGCTCGACATCCTCA<br>AGATGGCCACCGCCTACATCCGCCGCATGTCCTGGGCTTCGTGCCT<br>CGCCTTCCACCGCGAGGAGAAAACCTACATCCACATCATCGACCAG<br>CGCACGCGACAGCCAGTCCAGACCAAGTTCTACACCGACGCGATG<br>GTCGTCTTTCACCACGTCAACGCCTACGAAGAGGACGGCTGCATCG<br>TGTTCGACGTCATCGCCTACGAGGACAACTCCCTCTACCAGCTCTT<br>CTACCTCGCCAACCTCAACCAGGACTTCAAGGAGAACAGCCGCCTC<br>ACGTCCGTCCCGACGCTCCGCCGCTTCGCGGTCCCCCTCCACGTCG<br>ACAAGAACGCGGAGGTCGGCACGAACCTCATCAAGGTCGCGTCGA<br>CCACGGCCACCGCCCTCAAGGAGGAGGACGGCCAGGTCTACTGCC<br>AGCCGGAGTTCCTCTACGAGGGCCTCGAGCTCCCGCGCGTCAACTA<br>CGCGCACAACGGAAAGCAGTACCGCTACGTGTTCGCCACCGGCGT<br>CCAGTGGTCGCCCATCCCCACCAAGATCATCAAGTACGACATCCTC<br>ACCAAGTCGAGCCTCAAGTGGCGCGAGGACGACTGCTGGCCCGCT<br>GAGCCCCTCTTCGTCCCGGCGCCGGGCGCCAAGGACGAGGACGAC<br>GGCGTCATCCTCTCGGCCATCGTCAGCACCGACCCCCAGAAGCTCC<br>CGTTCCTCCTCATCCTCGACGCGAAGTCGTTCACGGAGCTCGCGCG<br>CGCGTCGGTCGACGTCGACATGCACATGGACCTCCACGGCCTCTTC<br>ATCACGGACATGGACTGGGACACGAAGAAGCAGGCGGCCTCGGAG<br>GAGCAGCGCGACCGCGCCTCGGACTGCCACGGCGCCCCCCTCACCT<br>AG

ninaB	ATGGCCGCCGGCGTCTTTAAGTCGTTCATGCGCGACTTCTTCGCCGT<br>CAAGTACGACGAGCAGCGCAACGACCCGCAGGCGGAGCGCCTCGA<br>CGGCAACGGACGCCTCTACCCCAACTGCTCGTCGGACGTCTGGCTC<br>CGGAGCTGCGAGCGCGAGATCGTCGACCCGATTGAGGGCCACCAC<br>TCGGGCCACATCCCGAAGTGGATCTGCGGGTCGCTCCTCCGCAACG<br>GCCCCGGTTCGTGGAAGGTCGGCGACATGACCTTCGGCCACCTCTT<br>CGACTGCTCGGCGCTCCTCCACCGCTTCGCCATCCGCAACGGCCGC<br>GTCACCTACCAGAACCGCTTCGTCGACACCGAAACGCTCCGGAAG<br>AACCGCTCGGCCCAGCGGATCGTCGTCACCGAGTTCGGCACCGCCG<br>CCGTCCCTGACCCGTGCCACTCGATCTTCGACCGCTTCGCGGCCAT<br>CTTCCGGCCGGACTCCGGCACCGACAACAGCATGATCTCGATCTAC<br>CCGTTCGGCGACCAGTACTACACCTTCACGGAAACGCCGTTCATGC<br>ACCGCATCAACCCCTGCACGCTTGCCACCGAGGCCCGCATCTGCAC<br>GACGGACTTCGTCGGCGTCGTCAACCACACGTCGCACCCTCACGTG<br>CTCCCGTCGGGCACCGTCTACAACCTCGGCACGACCATGACCCGCT<br>CGGGCCCGGCCTACACGATCCTCTCGTTCCCGCACGGCGAGCAGAT<br>GTTCGAGGACGCCCACGTCGTCGCCACCCTCCCGTGCCGCTGGAAG<br>TTGCACCCCGGCTACATGCACACGTTCGGGCTGACCGACCACTACT<br>TCGTCATCGTCGAACAGCCGCTCTCCGTCAGCCTCACCGAGTACAT<br>CAAGGCGCAGCTGGGCGGCCAGAACCTTTCGGCCTGCCTCAAGTG<br>GTTTGAGGACCGACCCACCCTCTTCCACCTCATCGACCGCGTCAGC<br>GGCAAGTTGGTCCAGACCTACGAGTCGGAGGCCTTCTTCTACCTTC<br>ACATCATCAACTGCTTCGAGCGCGACGGCCACGTCGTCGTCGACAT<br>CTGCTCCTACCGCAACCCGGAGATGATTAACTGCATGTACCTCGAG<br>GCGATCGCCAACATGCAGACGAACCCTAACTACGCGACGCTCTTCC<br>GCGGCCGCCCGCTCCGCTTCGTCCTCCCCCTTGGCACCATCCCCCCG<br>GCGTCCATCGCTAAGCGCGGCCTTGTCAAGTCGTTCTCGCTCGCGG<br>GCTTGTCGGCCCCCCAGGTCTCGCGCACCATGAAGCACTCGGTTTC<br>GCAGTACGCGGACATCACGTACATGCCGACCAACGGAAAGCAAGC<br>GACGGCCGGCGAGGAGTCGCCGAAGCGCGACGCTAAGCGGGGCCG<br>CTACGAGGAGGAGAACCTCGTCAACCTCGTCACGATGGAGGGCTC<br>CCAGGCGGAGGCGTTCCAGGGCACGAACGGCATCCAGCTCCGCCC<br>TGAGATGCTCTGCGACTGGGGCTGCGAGACCCCACGCATCTACTAC<br>GAGCGCTACATGGGCAAGAACTACCGCTACTTCTACGCTATCTCCT<br>CGGACGTTGACGCCGTGAACCCCGGCACCTTGATCAAGGTCGACGT<br>CTGGAACAAGAGCTGCCTCACCTGGTGCGAAGAGAACGTCTACCC<br>GTCGGAGCCCATCTTCGTCCCCTCGCCGGACCCGAAGTCGGAGGAC<br>GACGGCGTCATCCTCGCCTCGATGGTCCTCGGCGGCCTCAACGACC

	GCTACGTCGGCCTCATCGTCCTCTGCGCCAAGACGATGACCGAGCT<br>CGGCCGCTGCGACTTCCACACCAACGGCCCGGTCCCTAAGTGCCTC<br>CACGGCTGGTTCGCCCCTAACGCCATCTAG

Table S2: Primers used in this study.

Primers	Sequences (5'-3')	Description
BCO-F	GAGCGAAGGAAGCCGCATCGAC	Amplification of *BCO* genes
BCO-R	ACTGCAGGCGTGGAGAACGGAA	
LDP1-*Eco*RV-BTS1-F	ACTCAAAACACGCACGCACGCAAGGATATCATGTCGCTGGACTGGTACGA	Amplification of *BTS1* (1568 bp)
LDP1-*Spe*I-BTS1-R	CGAGGTGAGACGGGGCGGAATCGTACTAGTTCAGACTTTGGGAAGCTCGT	
LDP1-*Eco*RV-HMG1-F	ACTCAAAACACGCACGCACGCAAGGATATCATGGTCTTTGCCACGTCGCC	Amplification of *HMG1* (4518 bp)
LDP1-*Spe*I-HMG1-R	CGAGGTGAGACGGGGCGGAATCGTACTAGTCTAGTCCTTGCCCTTGGTCA	
GPD-*Eco*RV-CARB-F	ACAACACCAGATCAGTCACAGATATCATGCGCCCGCTTGCACGT	Amplification of *CARB* (2666 bp)
P2A-CARB-R	AGGGCCAGGGTTCTCTTCGACGTCGCCAGCCTGCTTGAGGAGCGAGAAGTTGGTAGCGCCCGAGCCACCGCGCAGGTACATCACCAAC	
P2A-BTS1-F	CGACGTCGAAGAGAACCCTGGCCCTATGTCGCTGGACTGGTACG	Amplification of *BTS1* (1596 bp)
P2A-BTS1-R	AGGGCCAGGGTTCTCTTCGACGTCGCCAGCCTGCTTGAGGAGCGAGAAGTTGGTAGCGCCCGAGCCGACTTTGGGAAGCTCGTGC	
P2A-CARRP-F	GACGTCGAAGAGAACCCTGGCCCTATGGGCGGACTGGACTACTGG	Amplification of *CARRP* (2310 bp)
Thsp-*Spe*I-CARRP-R	AGGTGAGACGGGGCGGAATCGTACTAGTTCACAGCGCCTGCCACG	

Tnos-*Xba*I-RT14-F	TCATCTATGTTACTAGATCCTCTAGACAATCGTGTGAAGAGCGAAGG	Amplification of *RT14* promoter (1046 bp)
ERG10-RT14-R	GTACGACACGCGTGCTTACTCTAAGAGGAGGAAAGAAGGAGGAAA	
RT14-ERG10-F	TTCCTCCTTCTTTCCTCCTCTTAGAGTAAGCACGCGTGTCGTAC	Amplification of *ERG10* (1971 bp)
Thsp-*Spe*I-ERG10-R	GTGAGACGGGGCGGAATCGTACTAGTTTAGAGGCGCTGGATGACGA	
Tnos-*Xba*I-FBA-F	TGTCATCTATGTTACTAGATCCTCTAGACTCTGCTCTCGCTCGCTGT	Amplification of *FBA* promoter (1548 bp)
ERG13-FBA-R	GTCGAGGGAGCAGGCGACATTGTAGCTAGTTAGTGTTAGAAGTGAAG	
FBA-ERG13-F	CTTCACTTCTAACACTAACTAGCTACAATGTCGCCTGCTCCCTCGAC	Amplification of *ERG13* (2021 bp)
Thsp-*Spe*I-ERG13-R	GAGGTGAGACGGGGCGGAATCGTACTAGTTTACGCGACGTGCGGCTTGTC	
Tnos-*Xba*I-PGI-F	TGTCATCTATGTTACTAGATCCTCTAGATGGCCGTCTGCCATTTCATC	Amplification of *PGI* promoter (1550 bp)
IDI1-PGI-R	ACGCACGAGTGGTGCAAGACATGGTTCGTAGCGTGGTGAGTG	
PGI-IDI-F	CACTCACCACGCTACGAACCATGTCTTGCACCACTCGTGCGT	Amplification of *IDI* (1226 bp)
Thsp-*Spe*I-IDI1-R	AGGTGAGACGGGGCGGAATCGTACTAGTTCACATGCGGATGATGCTGCCC	
Tnos-*Xba*I-RT14-F	TCATCTATGTTACTAGATCCTCTAGACAATCGTGTGAAGAGCGAAGG	Amplification of *ERG10* expression cassette (3413 bp)
FBA-Thsp-R	CACAGCGAGCGAGAGCAGAGCGCGCACTTCTCTGCACTG	

Thsp-FBA-F	CAGTGCAGAGAAGTGCGCGCTCTGCTCTCGCTCGCTGTG	Amplification of *ERG13* expression cassette (3950 bp)
LDP-Thsp-R	TTGTGGGGGCCGTGTCGGACCGCGCACTTCTCTGCACTGC	
Thsp-LDP-F	AAGATGCAGTGCAGAGAAGTGCGCGGTCCGACACGGCCCCCACA	Amplification of *LDP* promoter (849 bp)
Blh-LDP-R	CCAGTCGATCAGCATGAGGCCCATCTTGCGTGCGTGCGTGTTTT	
LDP-Blh-F	AAAACACGCACGCACGCAAGATGGGCCTCATGCTGATCGACT	Amplification of *Blh* with its terminator *Thsp* (1318 bp)
PGI-Thsp-R	CGACGATGAAATGGCAGACGGCCACGCGCACTTCTCTGCACTGC	
Thsp-PGI-F	ATGCAGTGCAGAGAAGTGCGCGTGGCCGTCTGCCATTTCATCG	Amplification of *IDI* expression cassette (3181 bp)
Thsp-R	TCCTGTCAAACACTGATAGTTTAAACTGAAGGCGGCGCGCACTTCTCTGCACTGC	
SpCas9-F	GAAGATCTCCAGACGGACCTTGAGAA	Construction of Cas9 expression cassette (5856 bp)
SpCas9-R	GCTCTAGAACTGGATTTTGGTTTTAGG	
gTGL-F	CAATTGATCTGCGGCCATA	Construction of gRNA expression cassette (325 bp)
gTGL-R	TCGACCTGCAGGCATGCA	
G418-F	ATGGGCAAGGAGAAGACCCA	Amplification of *G418* (810 bp)
G418-R	CTAGAAGAACTCGTCGAGCATGAG	
NAT-F	ACCCTCGACGACACCGCGTA	Amplification of *NAT* (563 bp)
NAT-R	TAGGGGCACGGCATCGACAT	
RT-ACTIN-F	CCATCCTCCGTCTCGACCTC	89 bp

RT-ACTIN-R	CGTGGTCGTGAACGTGTAGC	
RT-BTS1-F	CAAGAGAGTCGTCGGGATGC	131 bp
RT-BTS1-R	TGGCCGAGTTGATCGTCTGG	
RT-CARB-F	TCGTCGAGATGAGCAGCGAC	125 bp
RT-CARB-R	CATCGACAGCTCGTAGTGCG	
RT-CARRP-F	TCCTCTGGAGCACCTTCAGG	119 bp
RT-CARRP-R	TGTGCCGGATGAGGTACGAG	

Notes: The designation "RT" within the primer names is for real-time PCR. Underlined sequences indicate restriction enzyme recognition sites.

Table S3: The genes used in this study.

**Genes**	**Name**	**Protein ID**
BTS1	geranylgeranyl diphosphate synthase	RHTO_02504
HMG1	hydroxymethylglutaryl-CoA reductase	RHTO_04045
ERG10	acetoacetyl-CoA thiolase	RHTO_02048
ERG13	hydroxymethylglutaryl-CoA synthase	RHTO_02305
IDI1	isopentenyl-diphosphate isomerase	RHTO_05138
CARRP	phytoene synthase	RHTO_04605
CARB	phytoene dehydrogenase	RHTO_04602
